# Supplementary material for: Are carbon emissions trading and green financial instruments synergistic? -Comprehensive quantitative research based on content analysis
Source: PLoS One. 2024 Mar 7;19(3):e0298601. doi: 10.1371/journal.pone.0298601 (PMC10919601; doi:10.1371/journal.pone.0298601)
Supplement: S1 Data — (ZIP) [file pone.0298601.s001.zip › Data/地级市Z_F工作报告.zip!/╔╜╬≈/╜·╓╨╩╨/└┤╫╘╣1⁄2╓┌║┼í╛┬φ┐╦╩2╛▌═°í┐_2009.docx]

各位代表：

现在，我代表市政府向大会作报告，请予审议，并请各位政协委员和其他列席人员提出意见。

一、2008年工作回顾

2008年，全市人民在市委的正确领导下，紧紧围绕“加快科学发展、构建和谐晋中”主题，全面实施赶超战略，认真落实打基础、调结构、重民生、创环境、谋赶超的各项政策措施，经济社会发展取得新进步。

经济平稳较快增长。全市地区生产总值567.8亿元，增长9.3%。粮食总产13.3亿公斤。规模以上工业增加值273.1亿元，增长12.6%。第三产业增长11.9%。财政总收入116.1亿元，增长31.9%，一般预算收入46.9亿元，增长14.4%。主要经济指标增速高于全省平均水平。

发展更加趋于协调。全社会固定资产投资331.2亿元，增长16.7%，服务业投资高幅增长。社会消费品零售总额204.6亿元，增长26.2%。进出口总额7.3亿美元，增长43.3%。万元GDP能耗下降、二氧化硫和化学需氧量减排均超额完成任务。林木绿化率提高1.2个百分点。市城区环境空气质量首次达到国家二级标准。安全生产持续好转，全年未发生重特大安全事故。

改革开放成效显著。国有企业、集体林权制度等各项改革统筹推进。引进签约项目106个，利用外资6989万美元，增长22%。市城区逐步与太原市城市规划、煤气供应、广播电视实现对接。

人民生活持续改善。城镇居民人均可支配收入13036.8元，增长20%；农民人均纯收入4776.9元，增长13.6%。

各位代表，过去的一年，喜事多、大事多、难事也多。我们举全市之力，扎实开展奥运安保工作，圆满举办了奥运火炬平遥传递活动；我们迅速反应，科学处置，成功化解了三鹿问题奶粉事件造成的不利影响；我们万众一心，全力支援抗击南方雨雪冰冻灾害，有力开展了汶川特大地震救援工作，充分展示了晋中人民顾全大局、勇于奉献的精神风貌；特别是四季度以来，我们采取积极措施，主动应对金融危机的冲击和影响，经济社会发展经受住了巨大的挑战和考验！

我向大会郑重报告：市二届人大五次会议确定的任务圆满完成！市政府向全市人民承诺的实事全部兑现！

一年来，我们做了以下主要工作：

（一）瞄准赶超目标，坚持以科学发展理念指导工作，全力推动经济又好又快发展

大力推进工业兴市战略，工业经济速度加快、效益提高。实施新型工业翻番计划，狠抓布局调整、园区建设、龙头培育和项目带动，完成工业项目投资191亿元，重点项目开工率86.7%，对工业经济增长的贡献率达到60%。新增销售收入亿元以上企业32户。市级主抓的七个工业园区销售收入占全市32%。工业经济效益综合指数提高21.5个百分点，新型工业化水平达到44%。

毫不放松加强“三农”工作。全年市级财政投向“三农”2.7亿元，增长61%，各项政策性补贴资金增长128%。积极发展现代农业，启动现代农业示范区和二轮优势农产品区域布局规划，优势农产品集中度进一步提高。蔬菜生产和肉蛋奶综合产量居全省第一，寿阳甘蓝、菜豆角、马铃薯被确定为奥运专供蔬菜。新增国家级龙头企业3户，发展农民专业合作社1187个。新建沼气用户4万户。新农村建设有序推进。

坚持把发展服务业作为调整经济结构的主攻方向,全面拉开发展布局。三晋国际商贸物流城、山西能源工业国际分销物流港、金利恒华北钢材物流中心建设积极推进。新上旅游项目43项，建设五星酒店2家。成功举办了晋中社火节、平遥国际摄影大展等活动，旅游综合收入增长24.5%。坚持旧城改造和新城开发并举，房地产施工面积349万平方米，完成投资45.7亿元。第三产业增速高于GDP增速2.6个百分点，对经济增长的贡献率达到36.2%。

持续加大城乡基础设施建设力度。积极启动北部新城规划建设。新开工市政重点工程116项，完成投资28.9亿元。污水处理厂全部建成，垃圾处理厂全部开工，新增供热面积430.3万平方米、燃气用户2.7万户。6个骨干水源项目建设顺利实施，电网结构更加合理。新增公路通车里程618公里，公路密度达到86公里/百平方公里，在全省领先。

进一步加强生态环境建设。深入开展“百企节能行动”，对76户重点企业实施强制性清洁生产审核，关停企业23户，淘汰落后设备27台（座）。大力推进“蓝天碧水”工程，率先启动汾河流域生态环境治理修复与保护工程。实施造林绿化六大工程，完成造林合格面积44.1万亩。铺开城市重点绿化项目177.6万平方米，市城区人均公园绿地面积增加2平方米。

（二）高度重视社会民生建设，最大程度地解决事关群众利益的突出问题，推进和谐晋中建设

全面发展社会事业。认真实施“五大惠民工程”，全部免除义务教育阶段学杂费，撤并中小学校133所，新增3个省义务教育标准化建设合格县，成立晋中职教集团。新农合实现全覆盖，参合率89.3%。县乡村三级卫生医疗机构基础设施达标率85%，城市社区卫生服务覆盖面达到80%。企业“五险”扩面征缴顺利推进，零就业家庭实现动态消零，特困救助和城乡低保制度逐步完善，城镇居民医疗保险顺利推开。深入推进“文化晋中”建设。设立科技进步奖和科技担保基金。举办多项重大体育赛事，我市运动员首次参加奥运会。

精神文明和民主法制建设不断加强。深入开展精神文明创建活动，加强未成年人思想道德建设，城乡文明程度进一步提高。积极开展法制宣传和法律援助，全民法制观念逐步增强。认真做好信访和人民调解工作，积极开展联动大接访，及时化解矛盾。加强社会治安综合治理，严厉打击违法犯罪，有力维护社会稳定。

外事、侨务、气象、防震、档案、文物、地方志、民族、宗教、广电、新闻出版、人民防空、国防后备力量建设等工作取得新成绩，老龄、妇女、残疾人、人口和计划生育、关心下一代等事业都有新进步。

（三）不断创优发展环境，积极打造全省最有竞争力的投资洼地和发展高地

集中精力创建“六个一流”环境。建立四级政务服务网络，率先在全省开通移动电话会议和电子政务办公系统，开通“百件实事网上办”专栏和12345便民服务热线，实行“宁静生产日”制度。积极建设金融生态环境，努力构建多元化金融服务体系，浦发、兴业两家股份制银行落户，农村信用社成功改制，发展27个商业性小额贷款公司，依法清欠6494.5万元。开展“四城联创”，大力实施城乡清洁工程和农村“创三优”活动，城乡环境大为改观。11个县（区、市）一次性通过省级卫生城市验收，我市进入省级卫生城市行列。

不断加强政府自身建设。坚持依法行政，从严治政，完善政府工作规则，推进管理创新。各级政府自觉接受同级人大及其常委会监督，积极支持政协开展工作，104件人大代表建议、意见和325件政协提案全部办理完毕。加强与工会、共青团、妇联等人民团体的联系。全面落实党风廉政建设责任制，煤焦领域反腐败斗争顺利开展。监察、审计工作力度进一步加大。

过去一年成绩的取得，得益于省委、省政府和市委的坚强领导，得益于各级各部门的尽职尽责，得益于全市人民的拼搏奉献，得益于历届党委政府打下的坚实基础。在此，我代表市政府，向全市工人、农民、知识分子、各级干部和离退休老同志，向大力支持政府工作的人大代表、政协委员、各民主党派、工商联、人民团体、无党派人士，向关心帮助晋中发展的中央、省驻地单位和驻地部队、武警官兵，向积极参与晋中建设的海内外朋友，表示衷心的感谢！

在肯定成绩的同时，我们也要清醒地认识到，对照科学发展观要求，与发达地区相比，用人民群众的期盼衡量，我市经济社会发展还存在一些突出矛盾和问题。拉动经济增长的“三驾马车”中投资的比重大，支柱产业中传统产业的比重大，整个经济运行中资源能源消耗大，基础设施建设和社会事业发展欠账大，环境保护和节能减排的任务大，安全发展的压力大，改革开放和自主创新的力度小，大项目、大企业、大集团的数量小，财政公共服务和保障的能力小。这些问题，根本上是经济发展方式还比较落后，发展的协调性还明显不足。同时，各级政府公共服务、社会管理和市场监管的职能发挥还不到位，特别是个别部门和干部存在作风不实、执行不力、效率不高的问题。

对此，我们将时刻保持清醒头脑，敢于正视问题，勇于化解矛盾，善于驾驭调度，勤于工作落实，绝不辜负全市人民的期望和重托！

二、2009年工作总体部署

2009年，是落实“十一五”规划的攻坚之年，是实现“保五争四”赶超目标的关键之年。做好今年的政府工作，我们面临着前所未有的挑战和困难。当前，国际金融危机影响继续蔓延，宏观经济形势和环境复杂多变。一季度以来，我市经济运行虽然出现农业生产稳定、投资继续加快、消费保持平稳等积极变化苗头，但面临的下行压力仍然较大。工业已持续11个月回落、6个月负增长，外贸出口大幅下降，企业生产经营十分困难，近半数规模工业企业停产半停产，下岗失业、返乡农民工数量增大，经济下滑带来的城乡居民就业、收入等社会民生问题逐渐显现。宏观经济形势和环境的重大影响与自身的结构性矛盾相互交织、双重挤压，使我们赶超发展的步履异常艰难。

面对严峻的挑战和繁重的任务，我们不但要增强忧患意识，把困难估计得更充分一些，把措施准备得更周密一些，努力把不利影响降到最低限度，更要增强机遇意识，化危为机，借机而上，力保经济平稳较快发展。我们必须坚定信心、增强动力，知难而进、排难而上！我们的信心和动力，来自于中央一系列扩大内需、促进经济增长的战略举措，来自于省委、省政府转型发展、安全发展、和谐发展的有力部署，来自于我市多年发展奠定的坚实基础，来自于各级政府对全市人民福祉的高度负责！

各位代表，我们肩负的使命重大、责任重大、任务重大，既耽误不得，更失误不起！我们将凝聚全市人民智慧，汇集社会各界力量，以更加开阔的视野、扎实的作风、有力的措施，努力开创跨越赶超的崭新局面！

今年政府工作要全面贯彻党的十七大和十七届三中全会精神，深入贯彻落实科学发展观，围绕赶超发展战略目标，紧扣加快科学发展、构建和谐晋中主题，实施“产业转型、开放提速、环境创优、民生改善”四大工程，落实保增长、调结构、上项目、促就业、保民生各项任务，努力保持经济平稳较快发展。

主要预期目标是：全市地区生产总值增长10％；规模以上工业增加值增长12％；全社会固定资产投资增长9％；财政总收入增长8％；一般预算收入增长5％；社会消费品零售总额增长15%；城镇居民人均可支配收入增长12%；农民人均纯收入增长8%。 [09:30:09]

约束性指标是：万元生产总值综合能耗下降5.6%；二氧化硫、化学需氧量排放量分别下降0.5%和3.95%；居民消费价格指数涨幅控制在4%以内；城镇新增就业2.81万人，城镇登记失业率控制在4.2%以内。

上述指标，是着眼于保增长目标和赶超发展要求提出的。综合分析全市经济社会发展存在的不确定性因素，完成有很大难度。但我们坚信，只要措施得当，落实有力，就有可能实现这一目标。

做好今年政府工作，要突出把握好四方面重点：

一要加快产业转型。抓住新一轮结构调整契机，按照“主攻二产、大兴三产、夯实一产”思路，加快改造提升传统产业，大力发展高新技术产业和服务业，淘汰落后产能，整合生产要素，转变经济发展方式，实现保增长和调结构、增效益相统一。

二要推进开放提速。继续解放思想，深化改革，扩大开放，用改革的办法、开放的思维破解发展难题，努力扩大招商引资、内需外贸，加快区域一体化进程。

三要致力环境创优。全力打造一流的政务环境、金融生态环境、法制环境、城乡基础环境、安全环境、生态环境和人才成长环境，让产业成长、让企业滋润、让百姓受益。

四要突出民生改善。坚持把保障和改善民生作为全部工作的出发点和落脚点，下大气力解决事关群众利益的突出问题,集中力量办好事办实事，共享改革发展成果。

三、2009年主要任务

今年，要着重抓好十方面工作。

（一）全力以赴抓项目，增强投资对经济增长的拉动作用

实施项目带动战略，严格领导包项目制、跟踪服务制和重大投资项目行政审批全程代理制，确保中央、省重大投资项目和市级重大战略项目建设。

抢抓机遇争资争项。认真研究和用足、用活、用好国家、省一系列扩大投资政策，积极争取一批带动作用大、投资效益好的重大项目，力争争资争项实绩超过全省平均水平。

抓好重点项目建设。加快市级70项重点工程建设，完成投资160亿元。集中力量抓好潇河生态综合治理、城区集中供热、平遥古城环城地带综合整治等重点项目建设。继续开展“工业项目推进年”活动，加快阳煤集团寺家庄煤业600万吨、榆次国电和瑞光电厂、介休安泰200万吨大型机焦项目和120万吨H型钢、介休路鑫30万吨粗苯精制、100万吨甲醇等项目建设进度，完成投资230亿元，全市工业项目新增产值100亿元。

扩大招商引资。紧盯世界500强、国内200强企业，放开煤化工、装备制造、房地产、旅游业、现代农业等投资领域，积极参加各类贸易洽谈会，通过小分队招商、专业招商、以商招商、以亲招商，力争引进项目60个，引进资金100亿元。切实解决好项目用地、用水、环境容量等瓶颈制约，促进项目落地。

扎实做好项目储备，加强项目建设调度。拿出专项经费，加大项目前期投入，精心策划包装一批重大项目，储备一个、成熟一个、启动一个。建立项目建设责任制，科学管理、严格考核，确保项目建设取得实效。

各位代表，投资是拉动我市经济增长的主动力。我们一定要抓好项目建设，不断扩大投资，增强拉动能力，确保全市经济发展不掉队、赶超目标不落空！

（二）大力推进工业兴市战略，保持工业经济快速增长

不遗余力促进企业正常运行。保企业就是保增长。千方百计帮助企业渡过难关，切实为企业克服困境和加快发展提供一切应该提供的服务，是各级政府唯此为大的事情！今年要开展“企业服务年”活动。一是多渠道筹措资金，加大财政对工业企业发展的宏观调控和专项支持。二是认真落实国家和省各项税收优惠政策，研究实施企业税费减免具体措施，落实社保基金征收和就业培训“五缓二降一补”措施，切实减轻企业负担。三是创新金融服务，建立政府、金融机构、担保机构与企业之间的长效合作机制，完善中小企业信用担保体系，帮助企业多方式融资，解决企业资金困难。增加财政对各级担保企业注资，对企业贷款进行贴息，引导鼓励企业联保融资、自我救助。四是制定积极的营销策略，全面开展促销活动，扩大地方产品消费需求。积极争取企业出口退税，努力扩大出口。五是做好对重点行业、企业、主要产品和重要增长区域的监测分析，组织和指导企业开展技术和管理培训。

做大优势产业。坚持新型工业化方向，推进传统产业新型化、新兴产业规模化。煤炭行业要开展“煤矿安全质量标准化达标年”活动，加强机械化矿井建设，加大煤炭企业兼并重组力度，加快组建晋中煤焦集团。焦炭行业要抓住市场调整机遇，新建和改造一批大机焦项目，努力开拓营销市场。电力行业要突出左权电厂立项建设重点，扩大装机容量。冶金行业要大力发展特钢、高碳铬铁和不锈钢板材，提升碳素产品级次。洗煤、玻璃器皿、玛钢等行业要突出技术改造升级重点，提升产业素质。煤化工行业要加快中煤集团、太原煤气公司及安泰大型煤化工项目建设。食品加工业要抓住全省实施“食品百项工程”机遇，培育牛肉加工、食醋酿造、小杂粮加工三个特色产业集群。医药行业要依托医药工业园，重点扶持安特制药、德元堂药业、山西天生制药等企业做大做强。装备制造业要发挥“中国纺机名城”的品牌优势和人才技术潜力，大力发展精密化、智能化、节能化新产品；积极推动与吉利集团、北汽福田合作，加快建设全省汽车生产基地；加快寿阳金牛煤机项目达产达效，实现煤机制造新突破。

加快工业园区建设。采取品牌整合、龙头带动、企业聚集、协会服务等措施，重点抓好市级主抓的七大工业园区建设，力争园区工业达到规模以上工业总量的35%以上。

大力发展循环经济。积极发展煤—电—化、煤—焦—化、焦—铁—钢等产业，做长产业链。继续抓好介休省级循环经济试点市与中煤九鑫、安泰、三佳循环经济示范园和企业建设。制定实施以改造和对接传统产业为主的循环经济发展规划。

（三）加快现代农业建设步伐，扎实做好“三农”工作

贯彻落实中央一号文件精神，加大“三农”扶持力度，今年市级财政预算投入3.5亿元，比去年实绩增长30%，新增部分主要用于农村公共社会事业和农业重点工程。要做好五方面的工作。

一是启动建设晋中现代农业示范区。围绕高产高效粮食、优质无公害蔬菜、优质特色干鲜果、健康养殖和观光休闲农业五大基础产业,完善农副产品加工和农业市场化、组织化、社会化服务四大现代农业支撑体系，大力实施农田水利基本建设和农业机械化、信息化、生态绿化四大基础工程。今年市财政安排资金3000万元、榆太祁平四个项目县安排资金2000万元，首批启动太谷北洸万亩设施蔬菜园区、榆次得天缘生猪标准化养殖基地、祁县古县万亩色酥梨标准化生产园区等20个核心示范项目，加快玉米丰产方、机械化保护性耕作等45个支撑性项目建设进度。

二是加强农业基础设施建设。加大中低产田改造力度，提升耕地综合生产能力，确保全市耕地和基本农田保护面积不减少、质量不降低，确保全市非农业建设用地量质占补平衡，确保粮食安全。深入开展水利基础设施建设攻坚年活动，全面铺开110项水利工程，重点推进寿阳松塔、左权泽城、和顺恋思、灵石石膏山和介休龙凤等水库、水电站建设。完成祁县子洪等7座水库除险加固工程和昔阳郭庄水库主体改造工程。开工太谷郭堡水库和6座小型水库的加固改造工程。实施国家重点片区和流域坝系水保工程，全面推进汾河清水复流工程。

三是提升农业产业化水平。扎实推进二轮优势农产品区域布局规划，扩大设施蔬菜、干鲜水果种植，大力发展健康养殖业，力争3年内奶牛养殖全部实现入园饲养。培育壮大龙头企业，抓紧龙海实业、平遥牛肉等十大企业建设和发展。积极培育发展产地市场，巩固扩大销地市场，扩大网上销售份额。鼓励发展保鲜储藏库建设，积极推广反季节销售。推行农产品市场准入和产地准出制度，全市64%以上耕地实现无公害生产，“三品”新认证30个以上。市级重点培育和扶持100个农民专业合作社。加快构建农业新型服务体系。

四是多渠道增加农民收入。全面落实政策支持、技能培训、就业转移等促进农民增收的措施。大力发展“农家乐”休闲旅游，促进农村服务业发展，开辟农民增收新途径。加大扶贫开发力度，完成84个整村推进项目，搞好“雨露计划”，培训贫困地区富余劳动力2800名，转移率达到70%以上。

五是扎实推进新农村建设。完成500个村“十有十个一”目标建设任务。继续实施城乡清洁工程和农村环境“创三优”工作，加快落实“五个全覆盖”，努力改变农村面貌、转变农村生产生活方式、改善生产生活条件，发展农村可再生能源6万户以上，其中发展沼气用户2.4万户，力争全部完成改厕任务。

（四）扩大服务业规模，增强消费拉动经济增长的协调性

大力实施服务业“3+7”发展战略，重点抓好文化旅游、现代物流和房地产业发展。

推动旅游产业上档升级。围绕建设中国旅游名城和国际旅游目的地目标，加快旅游规划修编，整合古城大院、生态休闲、工农业体验、红色教育、商务会展五大资源，抓好十大重点旅游项目建设，全面提升景区景点基础设施条件。推进自然生态产品开发，完善特色餐饮、宾馆饭店、特色工艺品、文化演艺等要素体系，加大宣传促销力度，扩大旅游市场，全年旅游经济总收入增长28%。

大力发展商贸物流业。围绕构建“全省经贸枢纽”目标，加快建设一批专业市场、物流中心和物流基地，重点抓好三晋国际商贸物流城、山西金利恒钢材市场、山西能源工业国际分销物流港、榆太祁寿等农产品批发市场建设项目。加强城乡市场体系建设，继续实施万村千乡、家电下乡、品牌产品下乡和新网工程。努力扩大外贸出口，组织企业参加各类展会，积极开拓新兴国际市场，新增备案登记出口企业20户以上。

加快房地产业健康发展。围绕建设宜居城市目标，抓住旧城改造与新城开发机遇，以节能省地、环保恬静为特色，有序推进保障性住房开发建设。落实房地产业扶持政策，降低普通住房交易门槛，培育二手房市场及住房租赁市场，优化房地产投资环境。

（五）加强城乡基础建设，加快城乡一体化进程

做好城乡规划工作。完成晋中市城市总体规划修编、晋中市城市发展战略研究、北部新城启动区城市设计、潇河生态公园规划以及核心区设计等重点规划项目。完成祁县等5个县新农村建设规划和全市228个新农村重点推进村建设规划。加大详细规划编制力度，设区城市建设用地范围内控规覆盖率达到50%以上。

加强市政工程建设。加快北部新城启动区建设。市城区重点抓好龙湖桥和纬四桥等10大类、23项工程建设。组织实施热电联供一期管网配套、城市排水两大管网建设改造工程，力争热电联供入网300万平方米，第二污水厂投入运行，生活垃圾处理厂规范运行。启动潇河生态公园、晋商公园建设，市城区绿化覆盖率和绿地率分别增长4个百分点，力争达到省级园林城市标准。县（区、市）完成9大类119项市政工程项目，县（区、市）垃圾处理厂全部竣工。加强城乡环卫基础设施建设和环境卫生监管，全面提升城乡环境卫生水平。

加强公路交通建设。推进汾平高速公路建设，启动和榆平、龙城两条高速公路建设，做好大寨至平定高速公路前期工作，开工建设太原环城高速太谷连接线、完成108线义棠至霍州段一级公路改造项目，力争开工建设榆次至邢台一级公路改造项目。新增公路通车里程200公里、高级次高级路面里程260公里，县乡公路改造400公里。抓好晋中客运总站及6个县级汽车站的前期和开工建设，完成6个乡镇客运站建设。

加强电网建设。重点抓好榆次北500kv、左权220kv和介休连福、左权松树坪、寿阳宗艾3个110kv输变电工程，完成祁县东观等3个220kv站、灵石南关等3个110kv站增容工程。

推进晋中太原同城化进程。加快发展与重型装备、重型汽车紧密关联的零部件、基础件产业，提升机械制造配套能力；加快规划培育医药、食品、新材料等优势互补、产业互动、技术共享的产业集群；加快大型物流项目和农副产品批发市场建设，努力形成服务省城、辐射全省的农副产品供应基地和大型商贸物流集散中心；努力在道路体系的对接和建设上实现更大突破。

各位代表，县域经济是我市发展的基础和优势，是加快城乡一体化的重点和关键。我们要进一步加强县域经济发展规划、指导和考核，加大土地、项目、财政、金融、基础设施建设等方面的支持力度，大幅提升县域经济综合实力，全力推进经济带和城市群建设！

（六）加大改革开放力度，完善有利于科学发展的体制机制

国企改革力争突破。积极稳妥搞好企业破产工作，争取完成晋华纺织厂、山西绵纶厂和山西轻机厂的政策性破产。加快国有企业改制，重点推进晋中建设集团、晋中药材公司等企业改制工作。做好格芙兰公司、金恒公司改制的前期准备工作。积极推进县（区、市）、市直部门国有企业改革工作。认真做好企业资产处置和职工安置工作，积极为退休和在职职工解决工资、医疗等拖欠费用。健全国有资产监管体系，强化对国有企业负责人的考核，确保国有资产保值增值。

加大农村改革力度。深化农村土地经营制度改革，推进农业适度规模经营。推广左权“庄园经济”模式，提高土地集约化水平。节约集约用地，依法公开出让的土地100%实行招拍挂。加快集体林权制度改革，年内基本完成灵石、祁县和市级试点乡镇主体改革任务。推进水务管理体制改革，整合涉水行政职能，强化行业涉水管理。推进水工程供水价格改革，建立农业水权制度，推行农民用水自治。统筹开展城镇户籍制度、支农资金管理、农村金融体制等改革。强化乡（镇）政府公共服务职能。

深化政府公共管理改革。落实好政府机构改革任务。建立公共财政体制，调整优化支出结构，重点支持公共事业发展。完善财政集中核算、政府采购和工程项目招投标制度，降低成本。加快事业单位人事制度改革。

进一步扩大对外开放。牢固树立“扩大开放才有出路”的观念，扩大对外交流与合作，构建大开放格局。认真落实国家和省关于对外开放各项政策措施，完善对外开放产业、财税等相关政策，明确投资政策和准入标准，扩大对外开放领域，提高对外开放成效。

（七）扎实做好节能降耗和环境保护，大力推进生态文明建设

强势推进节能降耗。实施“双百户企业”节能行动计划，以煤焦、电力、化工、冶金、建材等高能耗行业为重点，加大节能技术推广和应用，坚决淘汰落后生产设备和工艺装置。鼓励引导煤炭企业进入一、三产业领域，加快资源型企业转产、转型。积极稳妥推进资源性产品价格和环保收费改革。全年节能100万吨标煤以上，淘汰落后产能100万吨。

毫不松懈地加强环境保护。突出抓好建设项目环境管理、工业污染源深度治理、环保基础设施建设和改善农村环境质量四大任务。认真执行总量控制、“三同时”和“区域限批”制度，实行严格的污染减排责任制和问责制，实施强制清洁生产审核，加快燃煤电厂、焦化厂脱硫设施建设，洗煤、焦化、电力、建材、冶金、化工及造纸企业实现废水零排放，玛钢、玻璃器皿等企业实行清洁能源置换。继续开展燃煤锅炉专项整治。加强环境安全监管，有效防范和处置各类环境污染事故。力争提前一年完成二氧化硫、化学需氧量排放量“十一五”减排任务。

全方位开展造林绿化。加快发展现代林业，实施“双十一”造林绿化攻坚，抓好国家退耕还林、天然林资源保护、太行山绿化工程，推进省级通道绿化、城郊森林公园建设、平原绿化和村庄绿化，全面启动市级河流滩涂绿化、干果经济林、农田林网和速生丰产林建设。完成造林35万亩以上，林木绿化率增加1个以上百分点。加强森林资源管护，推进封山禁牧，坚持不懈抓好森林防火。

各位代表，节约资源和保护环境是我国的基本国策，关系人民群众切身利益。作为负责任的政府，决不能因为经济发展而浪费资源、破坏环境，一定要为子孙后代留下满目青山、碧水蓝天！

（八）全面创优发展环境，积极打造区域性环境品牌

开展争创“七个一流”环境活动。今年，要在政务环境、金融生态环境和人才成长环境建设方面取得重大突破。

优化政务环境。深化行政审批制度改革，大力清理不合理收费项目。加快行政审批和电子监察系统建设，健全完善市县乡村四级政务服务体系，建立重大项目全程代理服务制度，打造重大项目落地简捷、高效、优质的“绿色通道”。拓展12345便民服务热线服务范围。

培育金融生态环境。加快金融创新，加强银企合作，调动银行放贷积极性。积极引进股份制银行、证券、保险、信托、基金、租赁等金融机构在我市设立分支机构。深化农村信用社改革，推动地方金融机构健康发展。积极推进企业与个人征信体系建设。加快发展区域资本市场，支持企业抓住资本市场低迷的机遇，买壳、借壳上市，支持优质中小企业上市，支持企业发行各类债券融资。加快实施商业性小额贷款组织信息化服务系统项目，尝试通过发行信托、债券等扩大直接融资。抓好农村小额人身保险试点工作。

改善人才成长环境。构建人才、智力、项目相结合的柔性引才机制，采取咨询、讲学、短期聘用、技术合作、技术入股、合作经营、人才租赁等多种形式引进人才。制定重点企业、重点项目人才需求紧缺专业目录，逐步形成以企业为依托的聚才引智机制。鼓励引导本土院校人才到企业、社会中介机构兼职服务，加快智力成果转化。关心帮助人才成长，千方百计解决实际困难。

（九）全面加强以改善民生为主的社会建设，加快构建和谐晋中

千方百计扩大就业。全面落实积极的就业政策，强化就业服务，促进更多劳动力实现自主就业。今年力争创业培训3500人、创业就业8000人、下岗失业人员再就业1.04万人。大力扶持劳动密集型中小企业和服务业发展，充分发挥其就业主渠道作用。实施公益性岗位援助，对零就业家庭成员、“4050”人员及困难国企失业人员，通过开发公益性岗位进行“托底”安置。鼓励重点工程吸纳城镇未就业退役人员、企业失业人员和返乡农民工就业。鼓励和引导高校毕业生面向基层创业就业。加强失业调控和预警，实行企业裁员报告制度，鼓励困难企业少裁员、不裁员，确保就业局势稳定。

各位代表，就业是民生之本，直接关系改革发展稳定大局。我们必须把稳定和扩大就业放在经济社会发展的突出位置，构建大劳动格局，打好就业“组合拳”，千方百计保就业！

健全城乡社保体系。大力实施非公有制企业和灵活就业人员三年扩面行动计划，努力扩大保障范围，提高企业和农村参保比例，企业退休人员社区管理服务率达到70%以上。继续提高城镇居民医疗保险入保比例，推进事业单位养老保险制度改革，实现失业保险和工伤保险市级统筹，做好被征地农民就业培训和社会保障工作。提高城乡居民最低生活保障标准和农村“五保”集中供养率。全面落实军烈属和伤残退伍军人优抚政策，积极发展社会福利和慈善事业。

把教育放在优先发展的位置。推进义务教育经费保障机制改革，提高生均公用经费标准。积极开展创建义务教育均衡发展县（区、市）活动，整体推进“千校达标工程”，撤并中小学校100所。认真解决农民工、困难家庭子女上学问题。大力发展职业教育和成人教育，加快普及高中阶段教育。办好职业教育集团，推进产学研结合。高度重视并扎实抓好校舍安全工程建设，把学校建成最安全、最漂亮、最让家长放心的地方！

大力发展医疗卫生事业。深化医药卫生体制改革，加强农村卫生服务体系建设，县乡村三级医疗卫生机构实现“村覆盖、乡达标、县提高”目标，逐步提高新型农村合作医疗财政补助标准和筹资水平，门诊住院双统筹覆盖6个县（区、市）。加快社区卫生服务中心（站）建设，努力配齐基层医疗卫生机构医疗设备和专业医护人员，鼓励大医院与基层卫生机构“联姻”结对。加大各级计生服务机构建设力度。

积极发展文化体育事业。加快文化公共基础设施建设，新建“农家书屋”400个,加大乡镇文化站、新农村建设示范村文化室建设力度。积极推进文化体制改革，引导民营企业投资文化产业，壮大文化产业规模。强化文化、新闻出版和版权市场监管。加强物质和非物质文化遗产的保护和管理，严格文化和文物市场监管。搞好第三次全国文物普查。进一步做好档案工作。广泛开展群众性体育活动，大力实施“农民体育健身工程”，加快新农村体育场地建设。

进一步加快科技创新。重点实施“463”科技支撑工程，不断提升科技创新水平，在农科教结合和与本土院校产学研合作上取得突破。

全力维护社会稳定。引深“平安晋中”建设，坚持群防群治、综合治理，严厉打击各种刑事犯罪，进一步完善和创新社会治安防控体系和公共安全保障体系，全面提升保障公共安全和危机管理能力。严格落实信访责任制，完善矛盾纠纷排查调处机制，畅通群众利益诉求渠道，解决群众反映强烈的热点难点问题。

各位代表!越是困难的时候，越要关注民生。无论困难多大、财力多紧、任务多重，我们坚持富民优先的理念不会变，财力惠民的强度不会降，民生工程建设的力度不会减，尽最大的努力让广大群众安居乐业、幸福和谐！

今年，市政府要为群众办好十件实事：一是维护、加固和改造中小学校舍26万平方米。二是实现县乡卫生机构达标率85%，新建249所标准化村卫生室。三是城镇居民基本医疗保险参保人数达到70%；将9.58万农村特困群众纳入低保范围，对1.59万农村“五保”对象实行应保尽保，解决好1.5万重点优抚对象的就医问题；帮助2784名城镇就业困难人员实现就业；培训农村劳动力10万人，转移农村劳动力3万人。四是建设村通水泥（油）路和新农村街道硬化1200公里。五是解决好农村24万人的饮水安全问题，完成100个行政村自来水入户工程建设。六是发展农家店和新农村便民店400个、配送中心14个、放心粮油店90家。七是确保市城区空气质量二级以上天数稳定在300天以上，保持稳定达到国家二级标准；其余10个县（区、市）城区二级以上天数稳定在280天以上，保持稳定达到国家三级标准。八是开工建设17613套、140.9万平方米城乡保障性住房，改造350户农村贫困残疾人危房，逐步解决好农村困难群众、棚户区居民、城市低收入群体和农村贫困残疾人的住房问题；继续实施扶贫移民工程，完成移民搬迁6700人。九是改造城市小街巷道路70条，其中市城区完成20条。十是实施广播电视村村通工程，保证644个20户以上通电自然村通广播电视。

（十）坚持不懈狠抓安全生产，全力促进安全发展

把安全作为兜底工程，预防为主、综合治理，重拳出击、标本兼治，确保安全生产形势持续好转。

扎实开展安全生产和食品药品安全专项整治。深入开展国家安全生产年和省安全生产专项整治活动，全面实施“村（企）日、乡周、县半月、市每月”安全隐患排查治理，严厉打击非法违法行为。煤炭行业要预防“五大灾害”，完善“五大系统”，大力建设本质安全型矿井。非煤矿山尾矿库要按标准设计、施工、排放，注重现场管理。继续加强道路治超工作，积极构建依法治超长效机制。认真抓好消防、易燃易爆物品、山林、道路交通的安全工作，抓好商场、学校、娱乐场所等公共聚集地的安全防范，加大避让搬迁、旧村耕地恢复、新水源地建设和地质灾害治理，做到不留死角、不留盲区、不留问题，坚决遏制重特大安全事故发生。切实加强安全生产应急救援工作，完善体制机制，整合救援队伍，增加物资储备和装备投入，加快建设综合应急救援基地，特别是地震应急救援体系建设，提高突发事故处置能力。强化重大动物疫病防控。深入开展食品药品安全专项整治，实行严格的食品安全质量追溯制度、召回制度、市场准入和退出制度。严厉打击食品药品制假售假行为，让广大群众买得放心、吃得安全。

强化安全责任。企业法人要切实履行企业安全生产第一责任人的职责，健全安全生产制度，落实安全防范措施。加大安全生产资金投入，不断改善安全基础设施和安全生产条件。严格用工管理，实行强制性安全培训，全面落实全员持证上岗制度。强化政府安全生产监管责任，健全安全工作领导干部全员负责制，严格“一岗双责”制度，强化工作落实。

严格责任追究。实施严密的责任制度和严格的责任考核，健全安全监管、安全责任、安全宣教体系，实现安全生产监管常态化、制度化。严格落实省政府出台的各项安全生产制度，坚持过程控制与结果控制并重，实行“一季一通报，半年一考评，年终总考核”制度，重奖重罚。严格事故查处和事前问责。

各位代表，抓安全就是抓发展、抓稳定、抓民生、抓和谐。我们决不以牺牲人民生命、损害群众利益为代价，换取一时一域的发展，决不要带血的GDP，一定要为广大人民生命与财产撑起安全保护伞！

四、加强政府自身建设

卓有成效地推进经济社会赶超发展，是对各级政府领导水平和工作能力的检验。我们一定要切实加强自身建设，和全市310万人民携手，全力加快科学发展、推动跨越赶超！

努力提高科学发展能力。加强公务员队伍教育管理，深入开展学习实践科学发展观活动，始终保持开拓进取、奋发有为的精神状态，创新经济社会发展理念，在发展速度上力求赶超，走在全省前列，在发展路径上力求转型，提升发展层次，在发展格局上力求统筹，营造和谐环境。

努力提高依法行政能力。认真贯彻《国务院关于加强市县政府依法行政的决定》，大力推行行政执法责任制。完善行政绩效考核，强化行政问责机制。完善重大事项集体决策、专家咨询、社会公示与听证制度，不断提高政府决策透明度和公众参与度。自觉接受人大、政协监督，认真听取民主党派、工商联和无党派人士的意见。加强反腐倡廉建设，坚决纠正损害群众利益的不正之风，严肃查处各类违法违纪案件。强化审计监督、行政监察和行政层级监督，加大政府投资项目审计力度。厉行节约、勤俭办事，严格控制一般性财政支出，严禁以各种名义用公款出国（境）旅游，压缩因公出国考察、公务购车用车、会议、公务接待费用支出，将更多的资金用于发展经济和改善民生。

努力提高应对危机能力。转变工作作风，主动问政于民、问需于民、问计于民，敢于创新，大胆实践。建立工作推进机制，开展县（区、市）分组竞赛，营造争先争上的发展氛围。实施市政府月度经济分析、重点项目推进、安全生产例会制度，改进工作方法，狠抓工作落实。

各位代表，新的一年充满挑战和希望，新的任务艰巨而繁重。让我们在市委的坚强领导下，深入贯彻落实科学发展观，坚定信心，迎难而上，勇于创新，全力以赴，以赶超发展的实践成果，向全市人民交上一份满意答卷！
